# Titanate nanoribbon-based nanobiohybrid for potential applications in regenerative medicine†

**DOI:** 10.1039/d2ra04753e

**Published:** 2022-09-21

**Authors:** Lionel Maurizi, Vanessa Bellat, Mathieu Moreau, Emmanuel De Maistre, Julien Boudon, Laure Dumont, Franck Denat, David Vandroux, Nadine Millot

**Affiliations:** Laboratoire Interdisciplinaire Carnot de Bourgogne, UMR 6303 CNRS/Université Bourgogne Franche-Comté 9 Avenue Alain Savary BP 47870 21078 Dijon France nadine.millot@u-bourgogne.fr; Société NVH Medicinal Dijon France david.vandroux@nvh-medicinal.com; Molecular Imaging Innovations Institute, Department of Radiology, Weill Cornell Medicine 413 E 69th Street New York NY 10021 USA; Institut de Chimie Moléculaire de l'Université de Bourgogne, UMR 6302 CNRS/Université Bourgogne Franche-Comté 9 Avenue Alain Savary BP 47870 21078 Dijon France; Haemostasis Unit, University Hospital of Dijon Dijon France

## Abstract

Nanoparticles capable of mimicking natural tissues represent a major technological advancement in regenerative medicine. In this pilot study, the development of a new nanohybrid composed of titanate nanoribbons to mimic the extracellular matrix is reported. During the first phase, nanoribbons were synthesized by hydrothermal treatment. Subsequently, titanate nanoribbons were functionalized by heterobifunctional polyethylene-glycol (PEG) to graft type I collagen on their surface. Biological properties of this new nanobiohybrid such as cytotoxicity to cardiac cells and platelet aggregation ability were evaluated. The so-formed nanobiohybrid permits cellular adhesion and proliferation favoring fine cardiac tissue healing and regeneration.

## Abstract 2

Titanate nanoribbons functionalized by heterobifunctional polymer and type I collagen for cellular adhesion and proliferation. This new nanobiohybrid affected neither cytotoxicity nor platelet aggregation ability.

## Graphical abstract

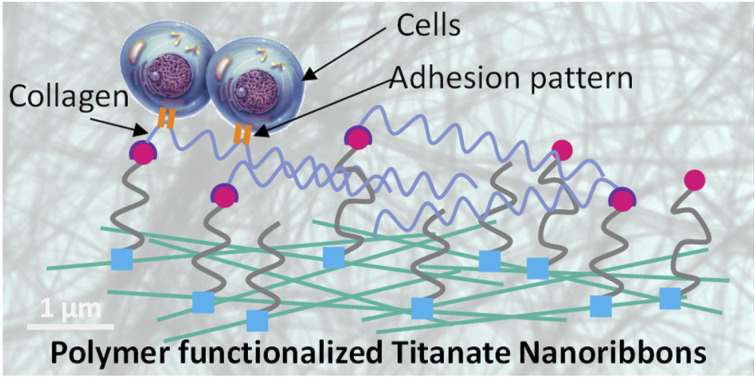

## Introduction

Recently, the market for tissue engineering has reached great heights, with key applications being clinical therapies and tissue modeling.^1–4^ Other applications of tissue engineering are personalized and regenerative medicine,^4^ cell-based biosensors *etc.*^5,6^ A market forecast of surpassing $4.8 billion is being predicted by the year 2028, for a total value of tissue-engineered products with upcoming technologies such as 3D bioprinting and artificial intelligence,^7^ decellularization and recellularization of organs and electrospinning.^8^

Regenerative medicine and tissue engineering aim to develop biological substitutes for the reconstruction of damaged tissues and the development of new healthy tissues. Currently, the main agents used for tissue regeneration are composed of proteins from the extracellular matrix (ECM).^9,10^ These molecules arrange themselves in a unique and complex 3D structure having structural and biological properties similar to tissue sources. Polymer networks are also used for tissue reconstruction and regenerative medicine.^1^ These structures enable fiber-based self-regeneration of tissue while limiting the risks of rejection and disease transmission strongly present in a transplant tissue from a healthy part of the body. Generally, these networks are made of poly nanofibers(lactic-*co*-glycolic acid) (PLGA),^11^ poly(l-lactic acid) (PLLA), poly(caprolactone) (PCL),^12^ poly(ethylene oxide) (PEO) also known as polyethylene glycol (PEG),^13^ poly(vinyl alcohol) (PVA),^14^ poly(ester urethane) urea (PEUU), gelatin, collagen, protein or fibrinogen.

The use of products based on biological components of human or animal origin, or polymer-based regenerative medicine or tissue reconstruction is not without risk. Those bio-products often cause side reactions, embolisms, allergies, infections, damage to nerves and tissue, transmission of diseases and viruses, the formation of blood clots, toxic and anaphylactic shock, necrosis of certain tissues *etc*.^15,16^

Commercial products made of polymers may generate, in turn, the compression of the nerves, tissue damage, inflammation, kidney and neurological damage, cerebrospinal fluids leaks, *etc.*^17,18^

Another approach has also been proposed to use inorganic fibers such as carbon nanotubes and nanofibers,^19,20^ TiO_2_ nanofibers or nanotubes^21,22^ or gold nanowires^23,24^ for tissue reconstruction and regenerative medicine.^25^ However, such structures are cylindrical in shape, with dimensions (length and width) of relatively low values.

A need therefore exists for a new material for use in regenerative medicine and tissue engineering which does not have the drawbacks listed above.

Since the observation of carbon nanotubes by Iijima in 1991,^26^ the tubular morphology has been thoroughly studied,^27^ especially those derived from titanium oxides.^28–30^ On the other hand, alternative bidimensional morphologies such as nanoribbons were rarely studied and were most often considered as undesired by-products of synthesis or as an intermediate step of the nanotubes formation.^31^ Nevertheless, for about fifteen-years, their specific properties have enabled titanate nanoribbons to become a full-fledged recognized nanostructure more and more studied by scientists.^32,33^

Titanate materials are of great interest in regenerative medicine due to their mechanical properties and high resistance to corrosion. Their surfaces are covered with hydroxyl groups that offers the possibility, compared to polymer-based products or biological components of human or animal origin, of functionalizing them with active molecules to couple, for instance, therapeutic and diagnostic effects^34^ for tissue regeneration. Furthermore, thanks to their radiosensitizing effect, titanates can also enhance any radiotherapeutic treatment.^35^ Elongated shapes of TiO_2_ nanomaterials such as titanate nanotubes (TiONTs) showed promising healing properties supported by a promotion of cell growth and proliferation thanks to TiONTs matrix.^36^ Furthermore, in implants, TiONTs or TiO_2_ are also providing photocatalytic activities enhancing their antibacterial properties.^37,38^ These properties can also reduce low prolonged inflammation reactions once implanted especially when coated with biopolymer such as collagen.^39^ Collagen has significant applications in tissue engineering. Owing to its excellent biocompatibility, biodegradability, facile extraction process, weak antigenicity and purification, scientific exploration concerning collagen have inspired the field of tissue engineering.^40^

In this pilot study, TiO_2_-based materials for regenerative medicine and tissue engineering were studied. Most particularly titanate nanoribbons (TiONRs) nanostructure with low cytotoxicity, functionalized with biocompatible active polymers and structural adhesion proteins (type I collagen) was developed. In this study, with a focus on cardiac damages, preliminary aggregation and adhesion measurements on fibroblasts demonstrated the particular interest of functionalized TiONRs to promote healing processes and regeneration of damaged heart's tissues.

## Materials and methods

### Chemicals

All chemicals and reagents were of analytical grade and used without further purification. Methoxy polyethylene glycol 5000 g mol^−1^ (mPEG_5000_ from Sigma Aldrich) and NHS-PEG_5000_-OH (JenKem Technology) were silanized to obtain mPEG_5000_-Si (see protocol SI_1) and NHS-PEG_5000_-Si respectively.

### Synthesis of TiONRs

The synthesis of TiONRs has been previously described.^41^ Briefly, titanate nanoribbons were synthesized by a hydrothermal treatment in strongly basic conditions. For this reaction, 110 mL of NaOH aqueous solution at 10 mol L^−1^ was prepared and introduced into a sealed Teflon reactor. Then 440 mg of TiO_2_ precursor (P25 Degussa) was added to the solution and the mixture underwent pulsed ultrasound treatment for 30 min at a power of 375 W (Sonics Vibra-Cells). The hydrothermal treatment took place at 180 °C with an autogenic pressure (7 bar), for 20 hours and under low mechanical stirring (150 rpm). The precipitate obtained at the end of the reaction was separated from the synthesis supernatant by a centrifugation cycle of 10 min at 11 000 × *g*. Finally, in order to wash the powder and to reach a neutral pH, the precipitate was dialyzed against water (at 3.5 kDa MWCO) for several days before being freeze-dried prior to characterization.

### PEGylation of TiONRs

TiONRs' surfaces were functionalized with a mixture of mPEG_5000_-Si and NHS-PEG_5000_-Si (see ESI and Fig. SI_1† for the silanization of PEG derivatives). 10.6 mg of naked TiONRs were dispersed under manual agitation in dichloromethane. Then 32 mg (mass ratio PEG : TiONRs = 3 : 1) of a molar ratio of 95% of mPEG_5000_-Si (30 mg) and 5% of NHS-PEG_5000_-Si (2 mg) were added to the TiONRs suspension. The mixture was magnetically stirred (150 rpm) for 48 h at 20 °C under inert atmosphere. The excess of PEGs was then washed with centrifugation cycles in dichloromethane and PEGylated-TiONRs were finally freeze-dried before further use and characterization. Hereafter, these nanohybrids are referred to as TiONRs-PEG-NHS.

### Functionalization of TiONRs-PEG

Functionalization of TiONRs-PEG-NHS with collagen was performed *via* NHS ester–amine reaction. Briefly, TiONRs-PEG-NHS was suspended in PBS at 50 μg mL^−1^ with type I collagen (Horm from Nycodem) at 10 μg mL^−1^ at 20–22 °C for 30 minutes. The suspension was then washed twice by centrifugation in PBS (4000 × *g*, 2 min) and the nanohybrids (TiONRs-PEG-Coll-I) were resuspended in PBS.

### Characterizations of TiONRs nanohybrids

Transmission Electron Microscopy (TEM) characterization was performed using a JEOL JEM-2100F microscope operating at 200 kV (point to point resolution of 0.19 nm). One hundred nanoribbons were counted in order to calculate nanoribbons average dimensions.

Powders were analysed using a Discovery TGA-TA Instruments with an air flow rate of 25 mL min^−1^. A temperature ramp of 5 °C min^−1^ from 25 °C to 800 °C was applied.

Specific surface area (SSA) measurements were performed using a Micromeritics Tristar II apparatus. Samples were outgassed *in situ* at 100 °C under a pressure of 26 mbar for 15 h and the measurements were performed at liquid N_2_ temperature with N_2_ adsorbing gas.

Considering mass losses at different temperatures coupled to the SSA measurements, grafting rates of the two polymers were calculated.

Polymer silanization was followed by proton nuclear magnetic resonance (^1^H-NMR). ^1^H-NMR spectra of synthesized polymers were recorded on a Bruker AVANCE 300 spectrometer in deuterated chloroform (CDCl_3_) at 300 MHz and 293 K (see ESI†).

### Cytotoxicity evaluation

With a focus on cardiac damages, MTT cytotoxicity tests were performed on cardiomyocytes and fibroblasts (rat's primary culture) in contact with naked TiONRs or TiONTs. Dose effect of TiONRs was evaluated for 72 hours. On day 1, the cells in 24-well plates were incubated (37 °C, 5% CO_2_) with TiONRs suspensions at 2, 20 and 66 μg mL^−1^ for 24 h. This operation was repeated twice on day 2 and 3 to study the dose effect (48 h and 72 h). For a morphology comparison, titanate nanotubes (TiONTs: length: 150 nm and diameter: 10 nm) synthesized following protocols from previous studies^42,43^ were also incubated with fibroblasts in the same conditions and at the final concentration of 66 μg mL^−1^. After incubation, cells were rinsed twice with PBS at 37 °C and incubated for 1 h with 500 μL of MTT at 2 mg mL^−1^ in PuCK G+ cell medium. Finally, MTT solution was replaced by 500 μL of isopropanol solution with 0.1 mol L^–1^ HCl for 45 min at 37 °C before optical analysis at 570 nm. The experiments were run in independent triplicate to perform statistical analyses.

### Platelet aggregation

Platelet aggregation tests were performed on platelet-rich plasma (PRP) incubated with naked TiONRs. Blood samples were collected from volunteer donors into citrate 3.2% collector tubes (BD Vacutainer France). Tubes were centrifuged at 150 × *g* for 10 min to obtain PRP. The residual blood was further centrifuged at 2500 × *g* for 15 min to obtain plasma poor plasma (PPP). The chosen PRP chosen was coming from voluntary donors and was selected to have at least 350 × 10^6^ platelets per μL of plasma. 290 μL of PRP (>300 g L^−1^) were mixed with 10 μL of naked TiONRs to obtain final concentrations of 1, 10, 25, 50, 100 and 250 μg mL^−1^. The suspensions of TiONRs in PRP were preincubated for 20 min at 37 °C before the measurements. As a positive control, 10 μL adenosine diphosphate (ADP) at 5 μM was used to stimulate the aggregation of 290 μL of PRP. Aggregation was measured *via* thrombo-aggregometer (Ta8v from SD Medical). Another experiment was also performed to study the influence of naked TiONRs on platelet aggregation. 290 μL of PRP were mixed with 10 μL of naked TiONRs at final concentrations of 100 and 250 μg mL^−1^. 11 minutes after reactions in the thrombo-aggregometer, 10 μL of ADP were added to induce the aggregation. Intensities of aggregation were then measured with light transmissions set up at 0% and 100% for PRP and PPP respectively.

### Cell adhesion

Effects of TiONRs' functionalization with collagen on cell adhesion were measured. Type I collagen (Coll-I), naked TiONRs, TiONRs-PEG-NHS, naked TiONRs + 10 μg mL^−1^ of Coll-I and TiONRs-PEG-Coll-I were placed in wells of 96 wells plates (Maxisorp from Nunc) at 50 μg mL^−1^ of nanohybrids and 10 μg mL^−1^ of Coll-I for 2 h at 37 °C. Then, wells were washed twice with PBS and passivated with bovine serum albumin (BSA) at 30 g L^−1^ in PBS to avoid unspecific binding. The excess of BSA was also washed twice with PBS. MRC-5 cells (human pulmonary fibroblasts) were incubated in the wells with 40 000 cells per well/100 μL at 37 °C for 2 h. Then the wells were washed twice with PBS and the adhesive cells were fixed with 100 μL per well of cold methanol (−20 °C) for 10 min. The cells were coloured with 100 μL per well of crystal violet at 5 mg mL^−1^ in methanol for 10 min. The reactants were finally washed with water and the cells were dried overnight before dissolving the crystal violet in 100 μL per well of SDS solution (10 mg mL^−1^) and measuring the absorbance at 570 nm. The values of adhesion were compared to the cell adhesion on 10 μg mL^–1^ of Coll-I.

## Results and discussion

### Characteristics of TiONRs

**Fig. 1:**
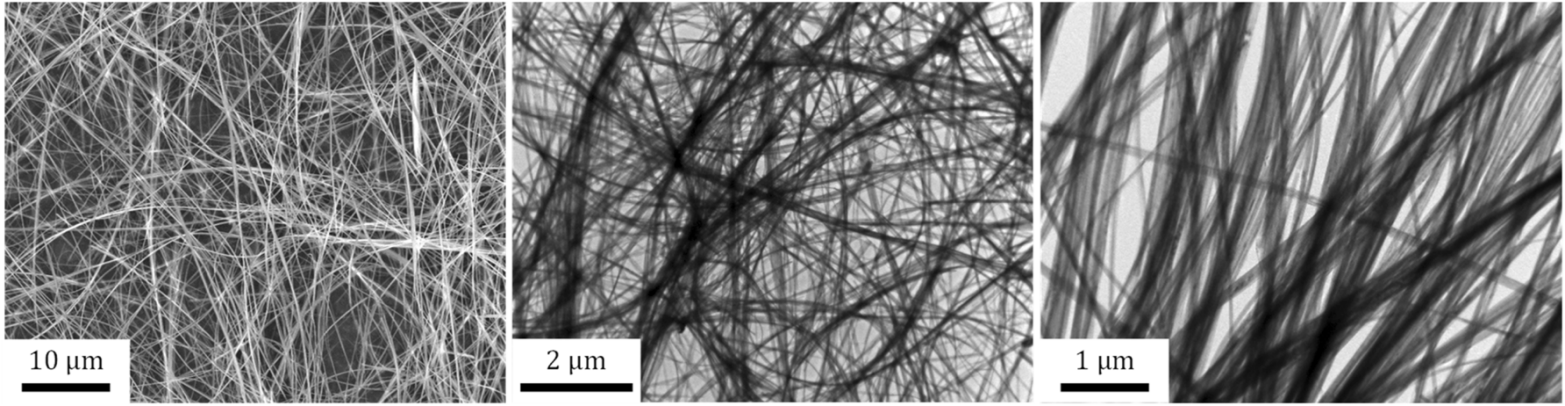
TEM pictures of titanate nanoribbons.

**Fig. 2:**
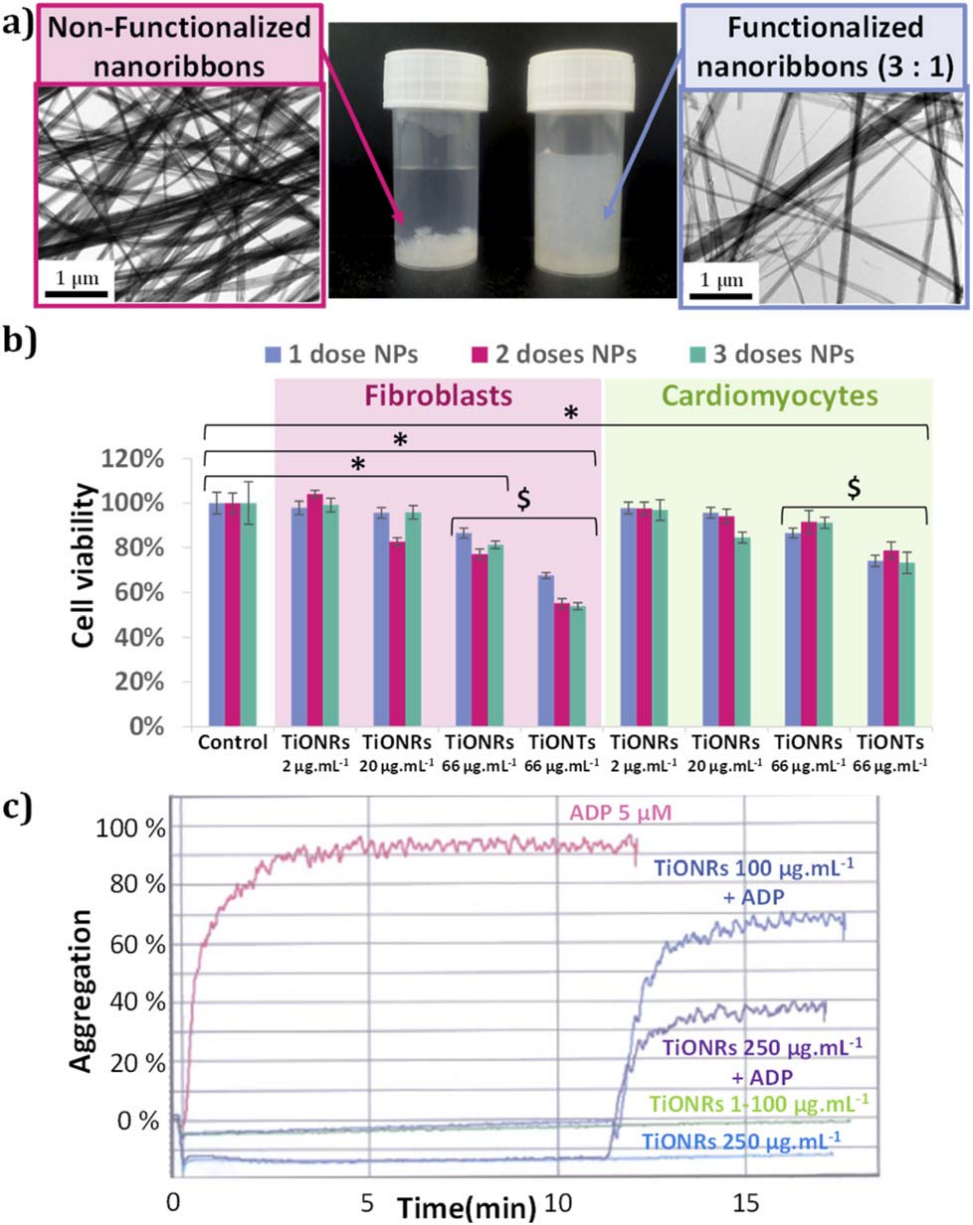
(a) Photograph and TEM pictures showing the dispersion of the naked and PEG-functionalized TiONRs; (b) cytotoxic dose effect of naked TiONRs and TiONTs at highest concentration on fibroblasts and cardiomyocytes. * Significant differences compared to the control *p* < 0.05; $ significant differences compared to the TiONRs *p* < 0.05; (c) aggregation's activation of platelets in presence of TiONRs at 1, 10, 25, 50, 100 and 250 μg mL^−1^ and ADP at 5 μM. ADP was injected 11 minutes after incubation. Platelets aggregation's activation in presence of TiONRs at 100 and 250 μg mL^−1^ was also measured. ADP: adenosine diphosphate.

The TiONRs obtained from hydrothermal syntheses have a specific morphology (Fig. 1) with a length between 1 to 20 microns, and a width varying from 70 to 200 nm. These average dimensions were measured on 8 reproducible batches of TiONRs (Fig. SI_2†). The average thickness of TiONRs is comprised between 3 and 40 nm; dimension's measurements was optimized in a previous publication.^41^ The morphology of the TiONRs is particularly suitable to highly and easily cover surface for potential tissue regeneration demonstrated by a good biomimetics with the extracellular matrix.^44^ By optimizing parameters, synthesis reaches 99% purity, that means less than 1% (in number) of by-products such as nanosheets, nanotubes or remaining TiO_2_ precursor are mixed with nanoribbons. This synthesis is reproducible as there are only few variations in terms of structure, morphology, and chemical composition (type Na_*y*_H_2−*y*_Ti_*n*_O_2*n*+1_, *x*H_2_O) after close to a dozen of syntheses (see ESI and Fig. SI_2†). Functionalization of TiONRs with Si-PEG-NHS improved the colloidal stability of TiONRs (Fig. 2-a). PEG polymers were chosen because of their high biocompatibility with many other nanomaterials.^42,45,46^ The success of the silanization by Si-PEG-NHS was confirmed with NMR analyses (Fig. SI_3†) proving a 90% yield of silanization and a final 80% yield of active NHS after functionalization. With a specific surface of 25 m^2^ g^−1^ for naked TiONRs coupled to the two mass losses (from 100 to 450 °C) of TiONRs-PEG-NHS (17.7% see Fig. SI_4†), corresponding to the degradation of mPEG_5000_-Si and Si-PEG_5000_-NHS, the concentrations of these two PEG on TiONRS are 1 and 0.1 molecules per nm^2^, respectively.

Thus, TiONRs have 0.08 active NHS per nm^2^. All the physicochemical characteristics of the TiONRs are summarized in Table 1.

**Table 1:** TiONRs physicochemical characterizations

	Length (μm)	Width (nm)	Thickness (nm)	Purity (%)	SSA (m^2^ g^−1^)	mPEG-Si (nm^−2^)	NHS-PEG-Si (nm^−2^)	NHS (nm^−2^)
TiONRs-PEG-NHS	[1–20]	[70–200]	[3–40]	99	25	1	0.1	0.08

### TiONRs as a potential nanohybrid for regenerative medicine

No significant cytotoxicities of TiONRs were found on cardiomyocytes. The maximum toxicity on TiONRs on fibroblasts was below 20% (Fig. 2-b) for the highest concentrations (66 μg mL^−1^) and no significant toxicity was found for the two other concentrations tested (2 and 20 μg mL^−1^). This value is more than twice lower than the cytotoxicity observed after incubation of 1 to 3 doses of TiONTs at a similar concentration on both cell lines. This low toxicity of TiONRs could be attributed to their original morphology. In fact, elongated nanomaterials such as nanotubes or nanorods have usually more chance to be internalized by cells and then to influence cell integrity and cause more damage. Regarding the TiONRs, because their lengths are higher than 1 μm and they are quite larger than classical nanotubes/rods (a few hundreds of nm compared to a few dozens of nm) they have less chance to be internalized and to promote cytotoxicity compared to the nanotube morphology that showed significantly higher cell killing effect.^47,48^ Furthermore, a dose effect was observed with TiONTs when none was observed after 3 successive doses of TiONRs.

**Fig. 3:**
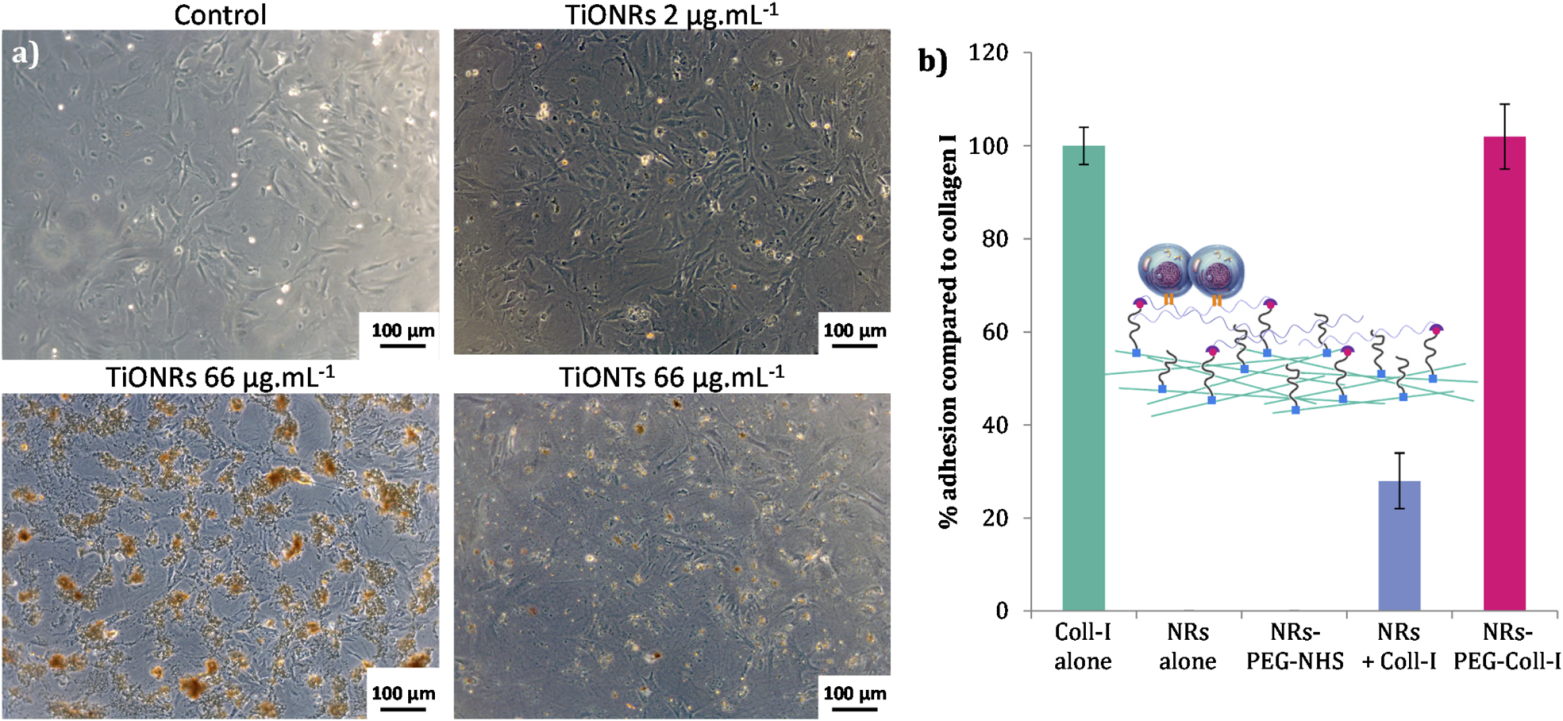
(a) Optical microscopy images of cardiac fibroblasts incubated with 2 doses of TiONRs at different concentrations and TiONTs at 66 μg mL^−1^. (b) Adhesion of fibroblasts on surface covered with type I collagen (Coll-I), naked TiONRs, TiONRs-PEG-NHS, naked TiONRs in presence of Coll-I and functionalized TiONRs-PEG-Coll-I. Concentrations of Coll-I (10 μg mL^−1^) and TiONRs (50 μg mL^−1^).

To confirm the hypothesis of lower cellular interactions of TiONRs compared to TiONTs, cytotoxicity measurements were correlated with optical microscopy pictures of fibroblasts (Fig. 3-a) incubated with nanoparticles. Two similar doses of TiONRs at 66 μg mL^−1^ showed much more aggregates of particles on top of the cells than with 2 doses of TiONTs at 66 μg mL^−1^ confirming that cells might not have internalized many TiONRs compared to TiONTs.

For concentrations up to 250 μg mL^−1^, TiONRs did not induce any spontaneous aggregation of the platelets in the PRP compared to the ADP which induced a rapid increase of aggregation within 3 min (Fig. 2-c). At a relatively high concentration (<100 μg mL^−1^), TiONRs do not have any side effect on platelet aggregation which allows their use as matrix for regenerative medicine. However, at 250 μg mL^−1^, TiONRs seem to slow down the platelet activity, as evidenced by the 2-fold reduction of the measured aggregation compared to the control and the TiONRs at 100 μg mL^−1^. A concentration of 250 μg mL^−1^ of TiONRs seems to be too high and might affect platelet aggregation.

Cellular adhesion efficiency of fibroblasts in the presence of TiONRs with or without PEG and functionalized or not with Coll-I was then quantified (Fig. 3-b). Surface covered with Type I collagen was used as a positive control. Coll-I is the most abundant collagen in human body made of fibers used to heal wounds and already well used to improve cellular adhesion in regenerative medicine.^49,50^ Among the non-toxic concentrations, a TiONRs' concentration of 50 μg mL^−1^ was chosen to cover the whole plate, thus limiting modulation of the platelet aggregation and forming a monolayer (the optimization of which was corroborated by optical microscopy: see Fig. SI_5†).

Surface coated with naked and PEGylated TiONRs do not allow cellular adhesion. 27% of adhesion were observed on naked TiONRs mixed with Coll-I. As TiONRs are completely covering the plate, this adhesion could certainly be explained by non-specific adsorption of the collagen on TiONRs surface allowing some cells to stick on the plate surface. Such unspecific adsorption is difficult to control and very common with proteins and nanomaterials.^51,52^ For the chemically functionalized TiONRs-PEG-Coll-I, the percentage of adhesion is as good as the adhesion with only Coll-I alone, proving the efficacy of the grafting of collagen on PEGylated TiONRs. As PEG is a well-known polymer that prevents proteins adsorption^53,54^ and that is also used for its antifouling properties preventing cellular adhesion,^55,56^ the obtained rate of adhesion proved that PEG is not accessible on the surface of the plate. It also proved successful covalent functionalization of Coll-I on TiONRs-PEG-NHS that allowed successful adhesion of fibroblasts on well-plate. Besides, it can be noted that the Coll-I coating prevents the direct interaction of the TiONRs' scaffold with fibroblasts.

## Conclusions

In this study, a new nanobiohybrid favoring cellular adhesion and proliferation has been developed for *in fine* tissue healing and regeneration, especially on cardiac damages. In a pilot study, we were able to synthesize in a reproducible manner titanate nanoribbons (TiONRs) with biocompatible functional polymers and type I collagen. TiONRs showed low cytotoxicity against cardiomyocytes and cardiac fibroblasts with a minimal dose effect compared to nanotube morphology. At concentrations below 100 μg mL^−1^, TiONRs neither induced nor inhibit the platelet adhesion opening the way for their putative use as bandages to cover skin wounds. We demonstrated that well controlled functionalization of TiONRs could lead to future materials for regenerative medicine with potential theranostics' applications thanks to the interesting properties of titanate material.

## Author contributions

Study design: VB, DV and NM. TiONRs synthesis, functionalization and characterization: VB, MM, JB, NM and FD. Biological studies: VB, LD, EDM and DV. LM and VB analysed the data and prepared figures from the experiments. Writing – Original Draft Preparation: LM; Writing – Review & Editing: LM, VB, NM, JB, FD, LD, EDM and DV; Funding Acquisition: NM and DV.

## Conflicts of interest

The authors declare no conflict of interest.

## Supplementary Material

RA-012-D2RA04753E-s001
