# Supplementary material for: Titanate nanoribbon-based nanobiohybrid for potential applications in regenerative medicine
Source: RSC Adv. 2022 Sep 21;12(41):26875–81. doi: 10.1039/d2ra04753e (PMC9490774; doi:10.1039/d2ra04753e)
Supplement: RA-012-D2RA04753E-s001 [file RA-012-D2RA04753E-s001.pdf]

Electronic Supplementary Material (ESI) for RSC Advances.
This journal is © The Royal Society of Chemistry 2022

1. Supplementary information

1.1 Synthesis of silanized and heterobifunctional PEGs

Methoxy polyethylene glycol 5000 g.mol^{-1} (mPEG$_{5000}$) was silanized with 3-isocyanatopropyltriethoxysilane (IPTES) with a molar excess of 1.8 IPTES for 1 PEG in THF at 20°C, 700 rpm for 5 days under inert atmosphere[55,56]. After reaction THF was evaporated and the mPEG$_{5000}$-Si was precipitated in heptane, filtered and freeze dried before use.

Heterobifunctional PEG was prepared to be grafted on nanoribbons *via* silane groups and to provide, at the other end, reactive groups for protein interactions. To this end, the carboxymethyl ester of N-hydroxysuccinimide (NHS) chemical group was chosen to increase reactivity between NHS and proteins.

Commercial NHS-PEG$_{5000}$-OH (JenKem Technology) was silanized with (IPTES) following and updating a protocol found in literature[57]. Briefly, IPTES and NHS-PEG$_{5000}$-OH were mixed at a 5:1 molar ratio in CH_2Cl_2 at 40°C, 700 rpm for 5 days under inert atmosphere (Figure SI-_1). The NHS-PEG$_{5000}$-Si obtained with a yield of 80% was also freeze-dried before use and characterization.

Figure SI_1. Reaction to obtain bifunctional PEG$_{5000}$ with NHS ester and triethoxysilane groups (NHS-PEG$_{5000}$-OH: NHS ester functionalized PEG$_{5000}$).

1.2 *Study of the reproducibility of the synthesis of TiONRs by TEM and X-Ray Diffraction of TiONRs*

The structure of the nanoparticles was obtained using different characterization techniques. To measure chemical structures of TiONRs, X-ray diffraction (XRD) was realized on an INEL CPS 120 diffractometer with Cu Kα radiation (λ = 1.54056 Å). Determination of the peak positions as well as curve fittings were performed on Fityk.59 software

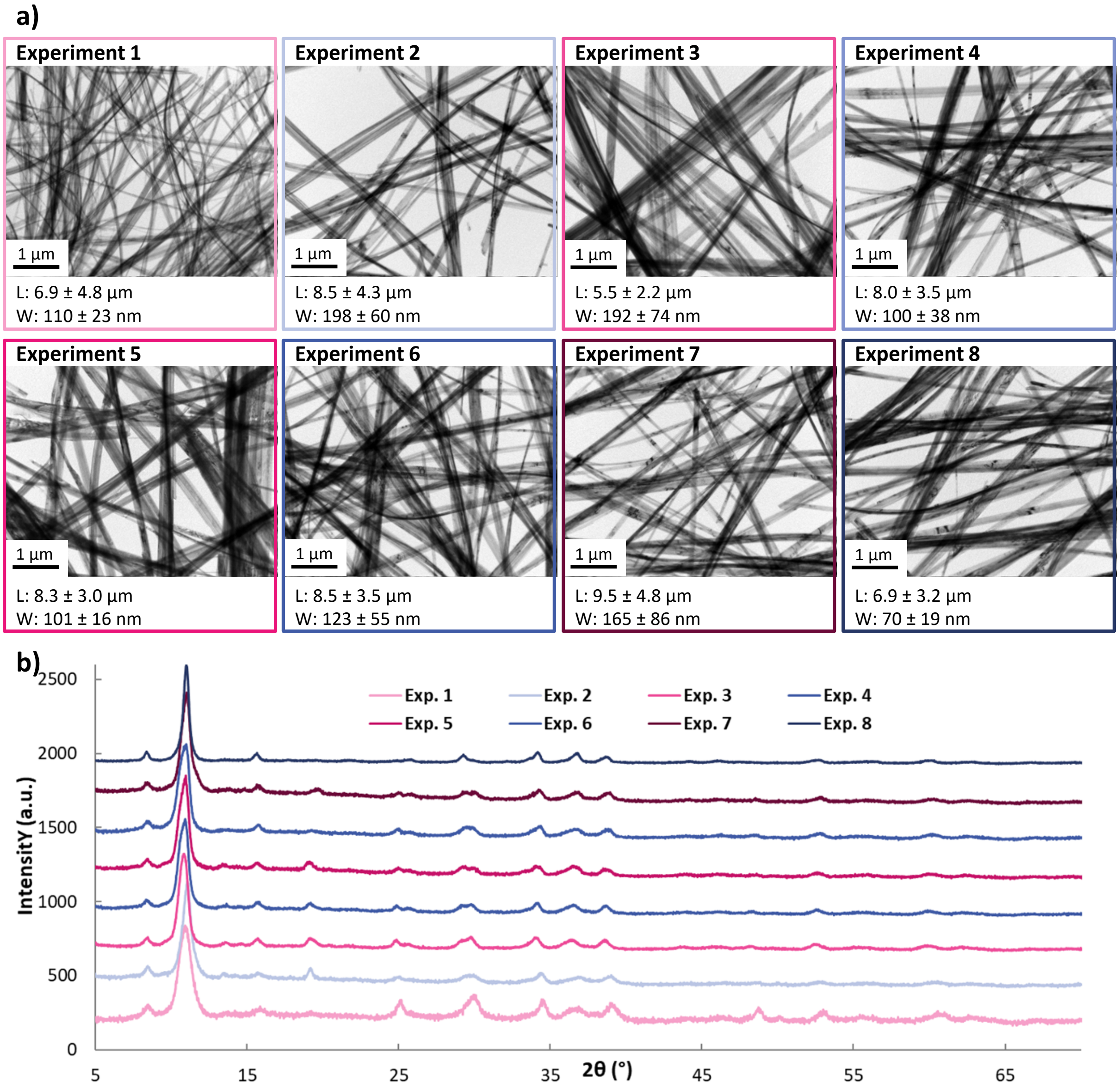

Figure SI_2 : a) TEM pictures of eight independent syntheses of TiONRs with characteristics showing the morphological reproducibility of the TiONRs (L: length, W: width measured on different TEM images at different magnifications) ; b) X-Ray Diffraction patterns of the same eight syntheses showing the chemical reproducibility of TiONRs

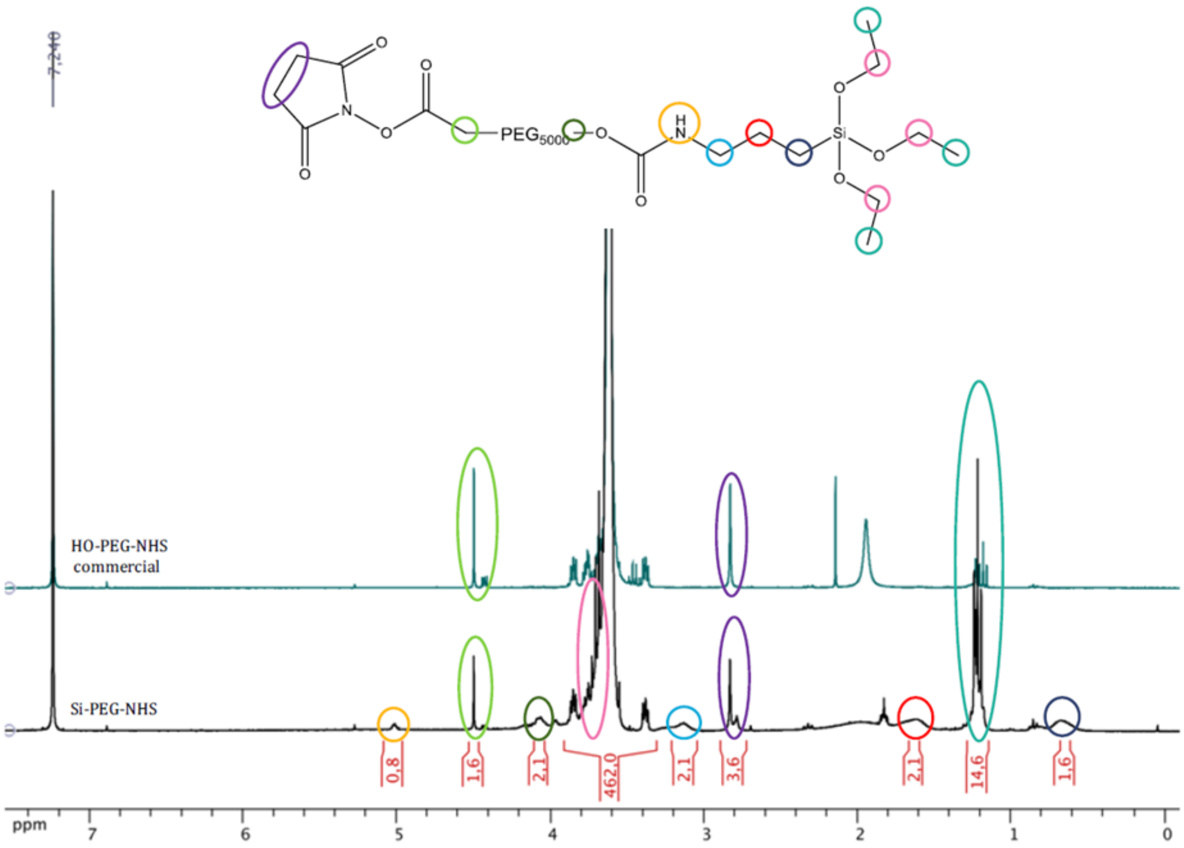

Figure SI_3 ^{1}H NMR Spectra of the bi-functional NHS-PEG$_{5000}$-Si (bottom) and starting NHS-PEG$_{5000}$-OH (top) (300 MHz, $CDCl_3$, 293K)

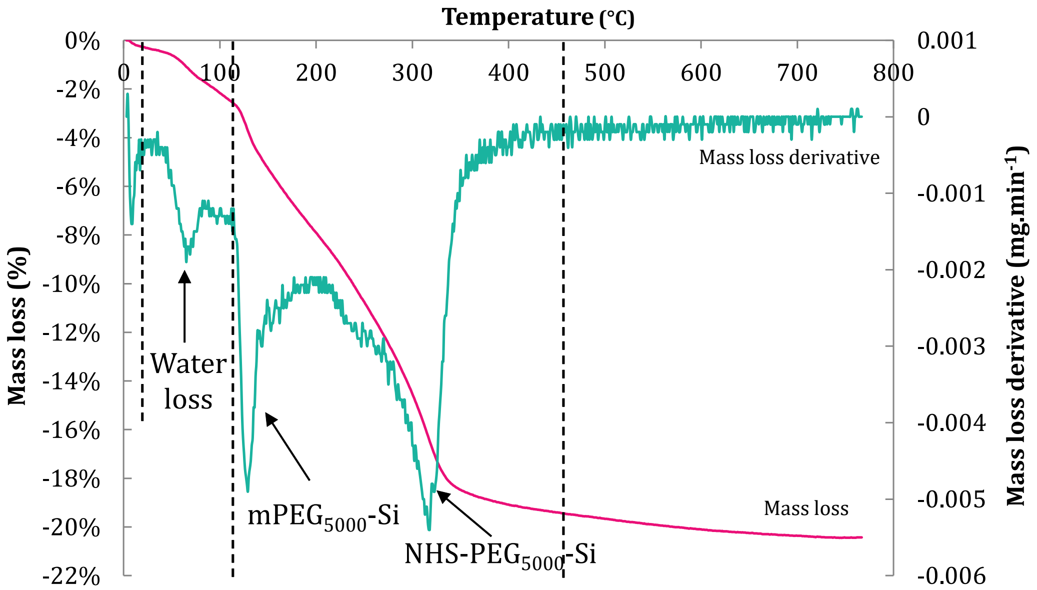

Figure SI_4. Thermogravimetric Analyses of PEG-functionalized Titanate nanoribbons

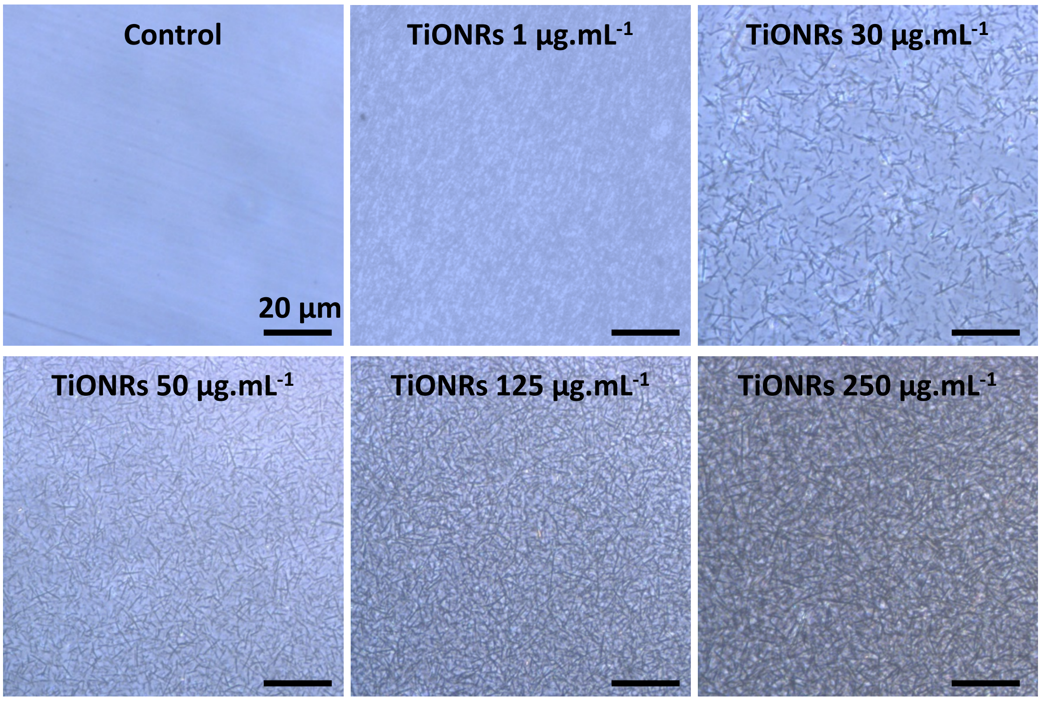

Figure SI_5. Optical microscopy images of cell culture plates covered with different concentrations of TiONRs. Below 50 μg.mL^{-1} the plate is not fully covered and above this value, there are multilayers of TiONRs. Scale bars: 20 μm
